# Differences in clinical and imaging presentation of maxillary sinus fungus ball with and without intralesional hyperdensity

**DOI:** 10.1038/s41598-021-03507-1

**Published:** 2021-12-14

**Authors:** Pei-Wen Wu, Ta-Jen Lee, Shih-Wei Yang, Yenlin Huang, Yun-Shien Lee, Che-Fang Ho, Chien-Chia Huang

**Affiliations:** 1 Division of Rhinology, Department of Otolaryngology, Chang Gung Memorial Hospital and Chang Gung University, No. 5, Fu-Shin Street, Kweishan, Taoyuan, 333 Taiwan, ROC; 2 Department of Otolaryngology—Head and Neck Surgery, Chang Gung Memorial Hospital and Chang Gung University, Keelung, Taiwan, ROC; 3 School of Medicine, Chang Gung University, Taoyuan, Taiwan, ROC; 4 Xiamen Chang Gung Hospital, Xiamen, People’s Republic of China; 5 Graduate Institute of Clinical Medical Sciences, College of Medicine, Chang Gung University, Taoyuan, Taiwan, ROC; 6 Department of Anatomic Pathology, Chang Gung Memorial Hospital and Chang Gung University, Taoyuan, Taiwan, ROC; 7 Department of Biotechnology, Ming Chuan University, Taoyuan, Taiwan, ROC; 8 Genomic Medicine Research Core Laboratory, Chang Gung Memorial Hospital and Chang Gung University, Taoyuan, Taiwan, ROC

**Keywords:** Diseases, Health care, Medical research

## Abstract

Maxillary sinus fungal balls (MSFBs) mostly occur in older individuals and demonstrate female predominance. Early diagnosis is important to avoid treatment delays. Intralesional hyperdensity (IH) indicates the presence of heavy metal deposition within fungal hyphae and has been the most specific characteristic of MSFB on computed tomography (CT). For those without IH on CT, the diagnosis of MSFB remains challenging. This study aimed to characterize clinical presentation of MSFB with and without IH and to study factors contributing to MSFB with no IH formation. We retrospectively identified 588 patients with MSFB. The clinical characteristics and CT findings were reviewed. Patients with unilateral MSFB had a mean age of 57.4 years and demonstrated female predominance (64.63%). The female-to-male ratio was highest at 51–60 years (2.02) and rose to 2.60 in MSFB with IH only. Compared to those with IH, MSFB without IH was significantly more common in males (OR = 2.49), in those with diabetes mellitus (DM) (OR = 1.87), adjacent maxillary odontogenic pathology (OR = 1.75). Complete opacification on CT was less common in MSFB without IH (OR = 0.60). Patients with MSFB without IH were more likely to have DM, no female predominance, adjacent maxillary odontogenic pathology, and partial opacification of the sinus, compared to those with IH. These may be helpful in better understanding of the formation of MSFBs without IH, early identification of them and prevention of post-operative recurrence.

## Introduction

Fungi are recognized as etiologic agents in a wide range of disease states of the nose and paranasal sinuses. The International Society for Human and Animal Mycology divides fungal rhinosinusitis (FRS) into invasive and non-invasive types based on histopathologic evidence of fungus penetrating host tissue^1^. The paranasal fungal ball (PSFB), usually found in the maxillary sinus (MSFB), is the most frequently encountered form of non-invasive FRS in clinical practice^1^. Several studies have reported an increasing incidence of PSFB over the past few decades in Asia^2–5^. Yoon et al. reported annual incidence of PSFB remained below 5% until 2001, but increased to over 10% since 2011 in Korea^4^. Liu et al. found out the incidence of PSFB in the last 5 years was significantly greater than that in the first 5 years during the 10-year study period (2008–2017) in China^5^. Studies have also shown that PSFB mostly occurs in older individuals, with a female predominance. The average age at presentation has been reported to be 49–61.1 years, with women accounting for 60.1–76.7% of cases^4–8^.

In addition, an increasing number of researchers have suggested that adjacent odontogenic infection increases the risk of MSFB owing to the close relationship between the antral teeth and sinus floor^9^. Furthermore, endodontic treatment on maxillary teeth is considered to be a significant risk factor for the development of MSFB^10^. Since maxillary odontogenic pathologies and endodontic procedures increase in frequency with aging, the role of odontogenic etiologies in MSFB may explain the predominance of this condition in the older population^6,11^.

Medical therapy has no role in PSFB treatment because of the side effects of systemic anti-fungal agents. On the other hand, endoscopic sinus surgery (ESS) for eradicating PSFB usually achieves good outcomes and has been considered as the standard treatment^7^. Therefore, early diagnosis of PSFB is important to avoid unnecessary medical therapy and treatment delays. Intralesional hyperdensity (IH), includes calcifications with a nodular or linear shape, indicates the presence of heavy metal deposition within fungal hyphae^12,13^ and has been the most specific characteristic of SFB on computed tomography (CT) scan images. In the literature, the prevalence of IH on CT images of MSFB ranges from 66 to 82%^5,8,14^. However, for those without IH on CT scan images, the diagnosis of MSFB continues to be a challenge. Although our previous study proposed an algorithm to improve the identification of MSFB based on the findings of pre-operative CT scans^15^, studies on the difference between MSFB with and without IH might be helpful for investigating pathogenesis of PSFB, especially for those without IH. In this study, we retrospectively investigated patients with PSFB who underwent ESS in our institute between 2005 and 2018. The aims of this study were to characterize clinical and imaging presentation of MSFB with and without IH, to compare the difference between them, and to study factors contributing to MSFB with no IH formation. These may be helpful in better understanding of the formation of MSFBs without IH, early identification of them and prevention of post-operative recurrence.

## Materials and methods

### Participants and setting

**Figure 1:**
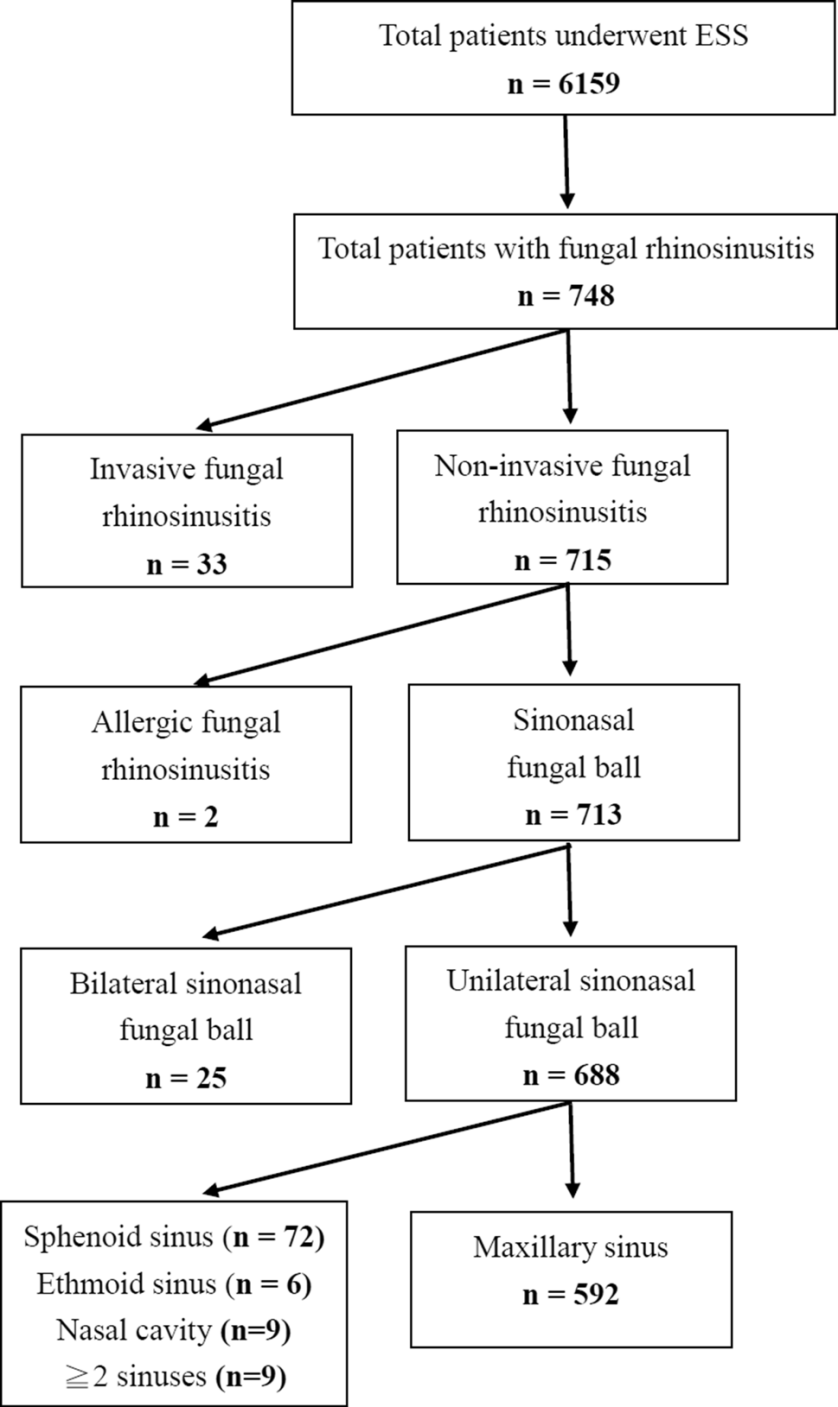
Algorithm for identifying study cohorts.

In our institute, every ESS will routinely send mucosal specimen for pathological analysis. Patients who underwent ESS for chronic rhinosinusitis and PSFB were identified using automated and manual retrospective chart reviews, as described below. We conducted an automated search of the histopathology database of our institute between 2005 and 2018. We then manually reviewed the chart records of the identified patients to confirm the study groups. A total of 6159 ESS procedures were identified during the study period; 713 patients with PSFB were selected from the database based on the exclusion criteria, which included patients with allergic fungal rhinosinusitis or invasive FRS (Fig. 1). In order to evaluate the incidence of PSFB, the ratio of the number of ESS procedures for PSFBs to the total ESS procedures was calculated for each year.

Since clinical features differ according to the location of the PSFB, and unilateral MSFB constituted the majority^16^, 592 patients with unilateral MSFB were identified for analysis. CT scan images of four patients were not available for review; we therefore evaluated the CT findings of 588 patients for the presence of complete or partial opacification and manifestations of IH. The maxillary sinus was assessed on three reconstructed planes (axial, sagittal and coronal) images of pre-operative sinus CT by window width of 2000 and window level of 0 for IH. IH was defined as spot or linear calcification (could be single or multiple) inside the maxillary lesion and was evaluated for each patient by two rhinologists independently. Total cloudiness of maxillary sinus without air was classified as complete opacification; otherwise as partial opacification.

**Figure 2:**
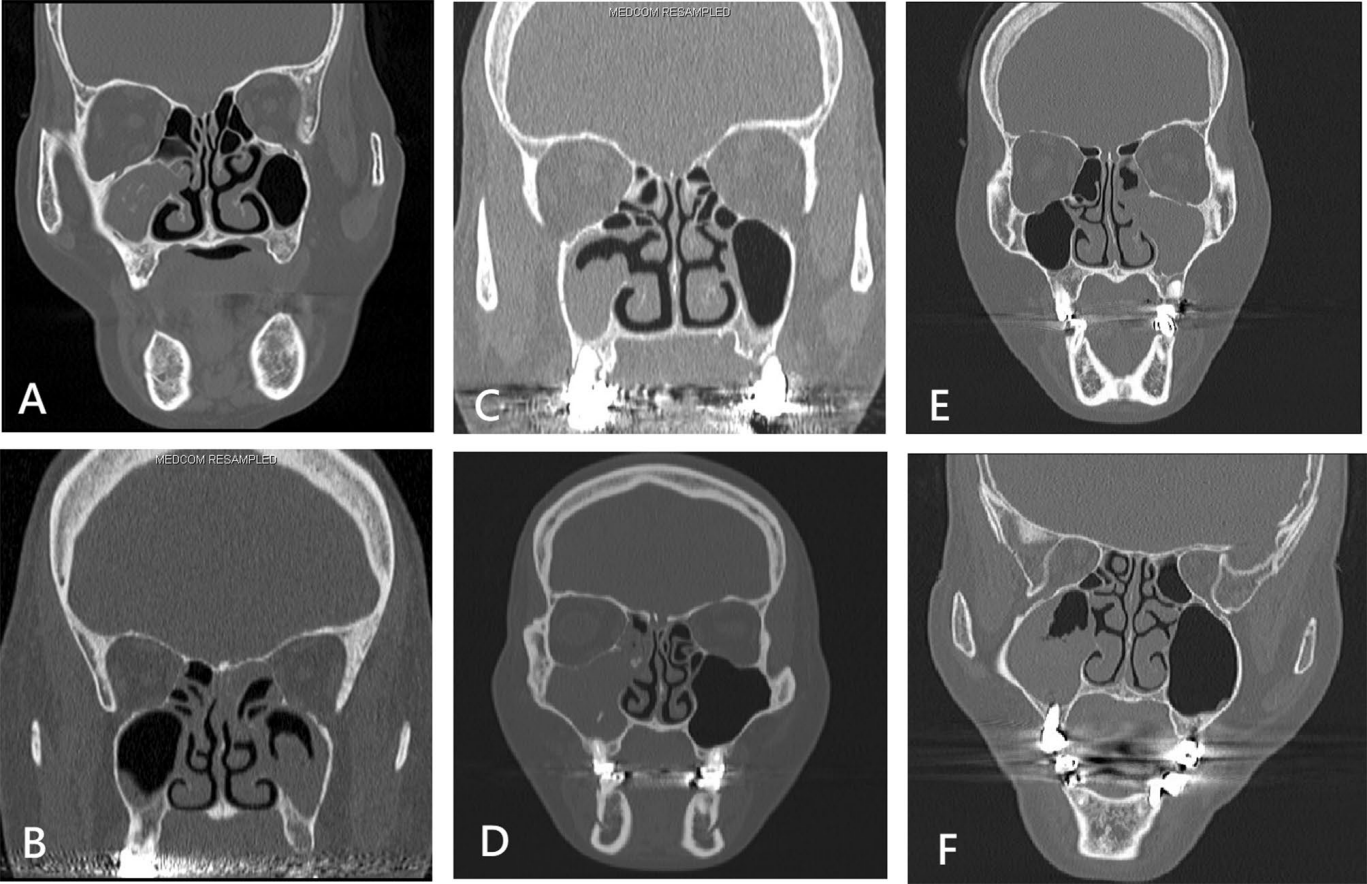
Computed tomographic features of maxillary sinus fungus ball with intralesional hyperdensity (**A**), without intralesional hyperdensity (**B**), with periodontal bone loss (**C**), with exogenous dental filling material (**D**), with periapical lucency (**E**), and with penetrating dental implant (**F**).

Odontogenic etiology was determined based on the clinical history and CT findings of adjacent maxillary odontogenic lesions including periapical lucency, periodontal bone loss, oroantral fistula, and exogenous dental reconstructive/restorative materials (Fig. 2)^17^. The study protocol was approved by the Chang Gung Medical Foundation Institutional Review Board (approval number: 202001450B0; protocol title: A retrospective analysis of patients underwent endoscopic sinus surgery at a single tertiary medical center in Taiwan (2004-2019). All research was performed in accordance with the relevant guidelines and regulations and all Institutional Review Board requirements. The requirement for informed consent was waived in view of the retrospective nature of the research and anonymity of the data.

### Statistical methods

Statistical analysis was performed with MATLAB 2015b program (MathWorks Inc., Natick, MA, U.S.A.) and SPSS ver. 26.0 (IBM Corp, Armonk, NY). Patients with MSFB were divided into two groups based on whether they presented IH on CT images; clinical characteristics and CT findings were compared between groups. Univariate analysis of categorical variables was performed using the χ^2^ test or Fisher’s exact test, where appropriate. Univariate analysis of continuous variables was performed using the unpaired t test. Logistic regression analysis was performed to evaluate the associations of multiple different variables with MSFB without IH. For each significant independent variable, the odds ratio (OR) and 95% confidence interval were calculated. A P-value of < 0.05 was considered to be statistically significant.

## Results

During the study period, the overall percentage of ESS procedures for PSFB in our institute was 11.58% (713/6159). The annual incidence of PSFB demonstrated an increasing trend since 2005, and has increased to over 10% since 2008. Twenty-five (3.51%) patients with PSFB had bilateral lesions. Among the unilateral FBs, single sinus involvement was observed in 679 (98.69%) cases; the maxillary sinus was involved in the majority of cases (86.05%). The distribution of sinus involvement by FBs is shown in Fig. 1.

The clinical characteristics of the 592 patients with unilateral MSFB are shown in Table 1. The mean age of patients was 57.4 (± 13.53) (range 18–90) years. Female predominance was seen in our series, with 380 female patients (64.63%) and 208 males (35.37%); 115 (19.56%) patients had comorbid medical conditions that may weaken the immune system, including diabetes mellitus (DM), liver cirrhosis, autoimmune disorders, end-stage renal disease, and bronchial asthma. Among them, DM was the most common comorbidity (14.80%). Recurrence was seen in 11 (1.87%) patients; 7 (1.19%) cases of complicated FB including facial cellulitis and orbital complications were observed. Fungal culture was performed in 211 cases (35.64%); among them, 29 (13.74%) showed positive results. The main species (14/29, 48.28%) was Aspergillus.

**Table 1:** Presentation of demographic, clinical, and therapeutic data of the patients examined.

Variable	Descriptive statistics
Case number	592
Age, year (mean ± SD)	57.44 (± 13.53)
**Gender**	
Male	208 (35.37%)
Female	380 (64.63%)
Odontogenic	119 (20.24%)
**Underlying diseases**	
Asthma	21 (3.57%)
DM	87 (14.80%)
ESRD	9 (1.53%)
Liver cirrhosis	5 (0.85%)
Autoimmune disease	7 (1.19%)
S/P maxilla surgery	10 (1.70%)
S/P maxillary dental implant	15 (2.55%)
NPC s/p CCRT	3 (0.51%)
Previous sinonasal cancer s/p CCRT	3 (0.51%)
Concomitant maxillary tumor	4 (0.68%)
Extrasinus complication	7 (1.19%)
Recurrence	11 (1.87%)
**CT image**	
Calcified spots	462 (78.57%)
Complete opacities	386 (65.65%)
**Laboratory data**	
WBC (1000/μL)	6.7 (± 1.8)
Positive fungus culture^a^	29 (13.74%)

*DM* diabetes mellitus, *ESRD* end stage renal disease, *S/P* status post, *NPC* nasopharyngeal carcinoma, *CCRT* concurrent chemoradiotherapy.

^a^Fungal culture was performed in 211 cases.

On the CT scan images, 386 cases (65.65%) showed complete opacification of the maxillary sinus, 462 cases (78.57%) had IH, and 119 cases (20.24%) had adjacent maxillary odontogenic pathology. The clinical characteristics of MSFB with and without IH were compared to evaluate the relationship between IH on CT, odontogenic pathology, and the degree of opacification; the results have been presented in Table 2. Multivariate logistic regression analysis with significant variables in univariate investigation showed complete opacification on CT was less common in MSFB without IH (OR = 0.60; P = 0.018). Compared to those with IH, MSFB without IH was significantly more common in males (OR = 2.49; P < 0.0001), in those with diabetes mellitus (DM) (OR = 1.87; P = 0.020) and adjacent maxillary odontogenic pathology (OR = 1.75; P = 0.022) (Table 3).

**Table 2:** Comparison of clinical characteristics between maxillary sinus fungal ball with and without intralesional hyperdensity on preoperative computed tomography scan images.

	MSFB with IH (n = 462)	MSFB without IH (n = 126)	P value^†^	95% CI
Age (years)	57.68 ± 13.49	56.53 ± 13.69	0.398	0.98–1.01
Gender (male)	142/462 (30.74%)	66/126 (52.38%)	< 0.001*	1.66–3.70
Odontogenic	85/462 (18.40%)	34/126 (26.98%)	0.045*	1.04–2.59
Opacification (complete)	313/462 (67.75%)	73/126 (57.94%)	0.045*	0.44–0.98
DM	61/462 (13.20%)	26/126 (20.63%)	0.047*	1.03–2.84
Asthma	17/462 (3.68%)	4/126 (3.17%)	1.000	0.28–2.60
ESRD	7/462 (1.52%)	1/126 (0.79%)	0.700	0.06–4.27

Data are represented as mean ± stand deviation.

*MSFB* maxillary sinus fungal ball, *IH* intralesional hyperdensity, *CI* confidence interval, *DM* diabetes mellitus, *ESRD* end stage renal disease.

*P < 0.05.

^†^Unpaired t test for continuous variables; χ^2^ test and Fisher’s exact test for categorical variables.

**Table 3:** Multivariate logistic regression analysis of the clinical characteristics related to maxillary sinus fungal ball without intralesional hyperdensity.

Variable	Odds ratio	95% CI	β	SE (β)	Wald χ^2^	P value
Gender (male)	2.49	1.66–3.74	− 0.91	0.21	19.21	< 0.0001*
Odontogenic	1.75	1.08–2.81	− 0.56	0.24	5.26	0.022*
Opacification (complete)	0.60	0.40–0.92	0.51	0.21	5.59	0.018*
DM	1.87	1.10–3.17	− 0.63	0.27	5.39	0.020*

*CI* confidence interval, *DM* diabetes mellitus.

*P < 0.05.

**Figure 3:**
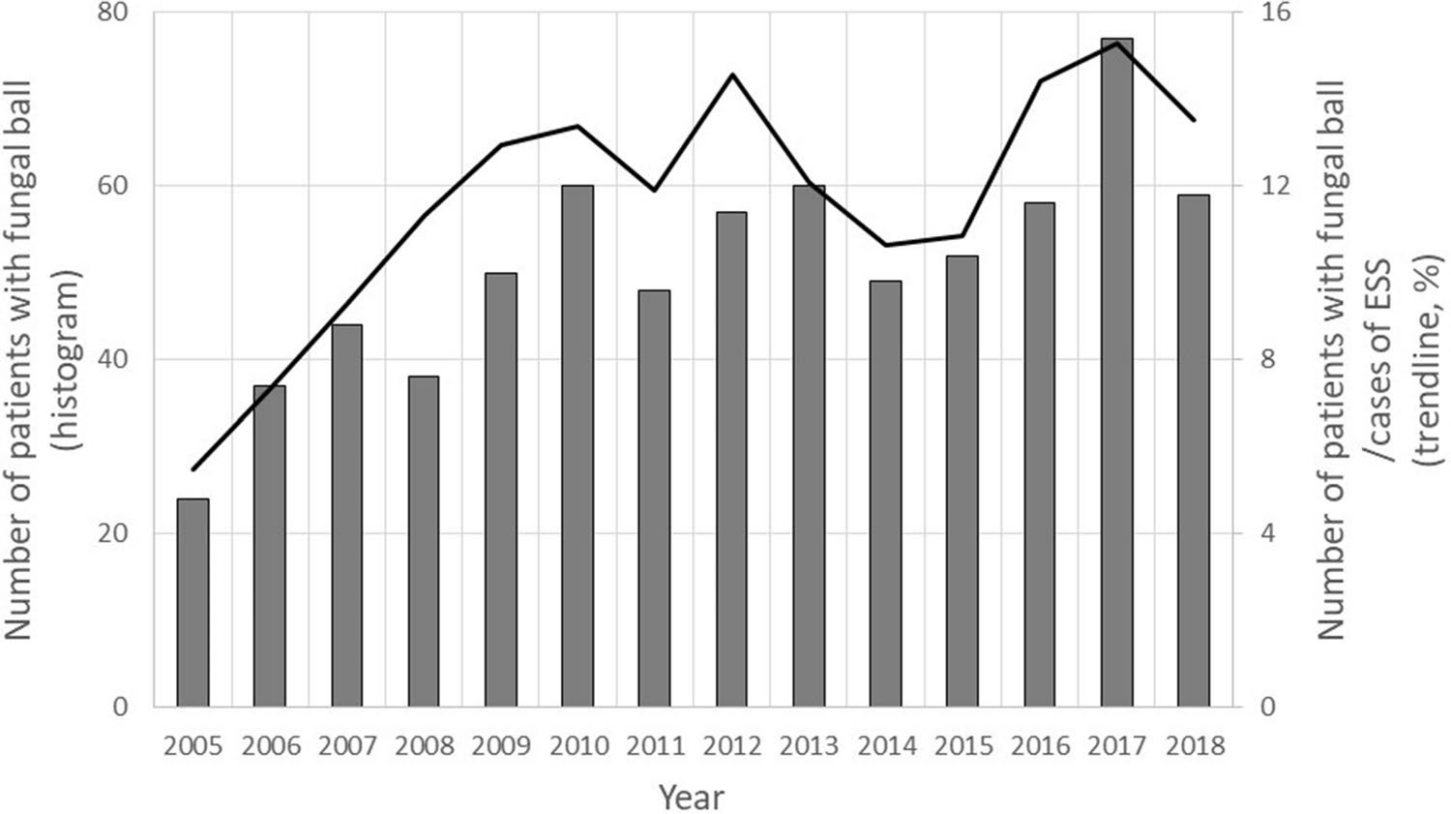
The incidence of paranasal sinus fungus ball (line chart), the ratio of the number of endoscopic sinus surgery (ESS) procedures for paranasal sinus fungus ball (histogram) to the total ESS procedures was calculated for each year and reflected an increasing tendency.

In order to evaluate the incidence of PSFB, the ratio of the number of ESS procedures for PSFBs to the total ESS procedures was calculated for each year and showed in Fig. 3. In 2005, the proportion of PSFB cases in the total number of cases of ESS was only 5.5%, but exceeded 10% since 2008 reflecting an increasing tendency.

**Figure 4:**
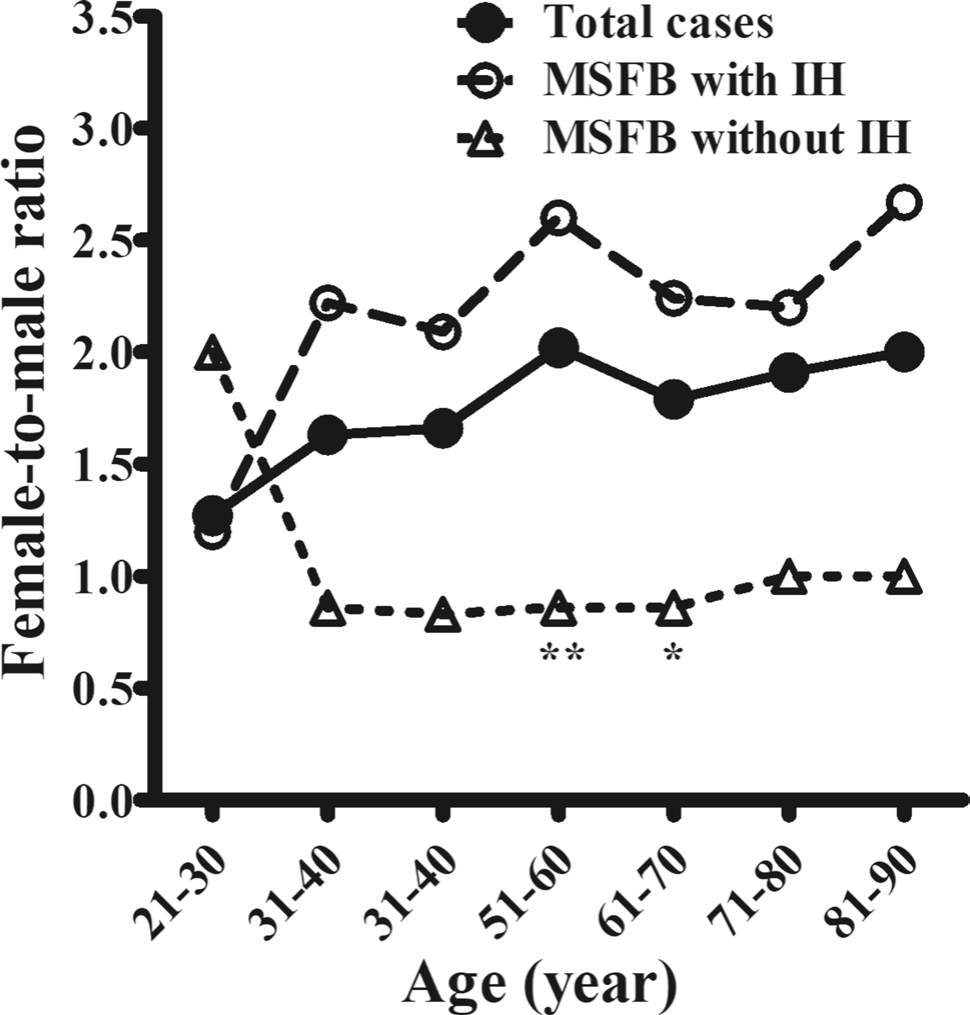
Female-to-male ratio in each decade of age. Female predominance was seen in the maxillary sinus fungus ball (MSFB) cases, overall. The female-to-male ratio was highest at 51–60 years (2.02) and rose to 2.60 in cases of MSFB with intralesional hyperdensity (IH) in the same range of age. In contrast, female predominance was not seen in patients without IH on CT scan images. The difference was significant in the 51–60 and 61–70 years’ groups. *P < 0.05, **P < 0.01.

The female-to-male ratio in each decade of age was further analyzed in the two study groups; the results have been shown in Fig. 4. Female predominance was seen in all MSFB cases. The overall female-to-male ratio was highest at 51–60 years (2.02) and the ratio rose to 2.60 in the MSFB with IH group in the same age range. In contrast, female predominance was not seen in the MSFB without IH group. The difference between the 51–60 and 61–70 years groups was most significant.

## Discussion

In this cohort, the female-to-male ratio was highest in the 51–60 years’ group, corresponding to the age of menopause. This may implicate the association of post-menopausal hormonal changes and the formation of PSFB, and explain the female predominance, especially in the elderly. Additionally, patients with MSFB without IH on CT images were more likely to have DM, adjacent maxillary odontogenic pathologies, and partial opacification of the sinus compared to those with IH; female predominance was not seen in this patient group.

Studies have shown that PSFB mostly occurs in older individuals and has a female predominance^5–8^. However, there is no consensus on the explanation for this phenomenon. Our study focused on unilateral MSFB; the impact of age and sex were analyzed. We observed that the female-to-male ratio was highest at 51–60 years of age (2.02). The mean age (SD) at menopause in Taiwan is 50.2 (± 4.0) years^18^. This suggests that post-menopausal hormonal changes may be associated with the formation of PSFB. Although the precise mechanism is not clear, the nasal mucosa is affected by changes in female sex hormones^19^. Özler et al. and Gumussoy et al. both reported on the prolongation of nasal mucociliary clearance times in menopausal women^20,21^. Impaired mucociliary activity may weaken the defense mechanism of the nasal respiratory epithelium, resulting in failed clearance of airborne fungal spores and formation of PSFB.

However, female predominance was not seen in the MSFB without IH group on CT scan images. In this group, patients were more likely to have DM and adjacent maxillary odontogenic pathologies. We hypothesized that both these factors contributed more directly to impair sinonasal ciliary function and increase the risk of local infection and/or inflammation in the maxillary sinus than sex hormones; the gender-related difference was therefore diminished in this group.

The PSFB is the most common FRS, but its incidence in the general population is unknown. Recent studies have shown that the incidence of PSFB has been increasing since the mid-2000s^2–5^. In this study, we analyzed the data of 713 patients with PSFB treated at a single medical center between 2005 and 2018. In 2005, the proportion of PSFB cases in the total number of cases of ESS was only 5.5%, but has exceeded 10% since 2008. Although these results may not represent the exact annual incidence of PSFB in Taiwan, they reflect an increasing trend. Improved awareness of the disease, improved diagnostic tools and techniques, broad-spectrum antibiotic use, and aging of the population may have contributed to this phenomenon^5^. Among these causes, we speculated that the extensive use of CT scans may have been the most important factor. National health insurance has paid in full for indicated CT scans in Taiwan since 1995. The database shows that the utilization of CT scans in Taiwan has increased between 1997 and 2008^22^. In addition, the popularity of dental implants increased the possibility of discovering asymptomatic MFSB during the pre-procedure evaluation.

In the current study, patients with MSFB without IH on CT scan images were more likely to have DM and adjacent maxillary odontogenic pathologies. Both these clinical features contributed to increased susceptibility to secondary bacterial infections. The clinical symptoms may be more severe, resulting in timely visits to healthcare facilities. We may consider patients with MSFB without IH on CT scan images to have early-stage MSFB. Short disease duration and inadequate fungal metabolic metal deposition resulted in partial opacification without IH of sinuses on CT scan images. These may be helpful in better understanding of the formation of MSFBs without IH, early identification, and prevention of post-operative recurrence. We should pay more attention to the comorbidities such as DM and odontogenic pathology in treating patients with MSFB without IH on CT scan images peri-operatively.

Unlike invasive FRS, PSFB usually occurred in immune competent patients. The relationship between PSFB and chronic disease has not been confirmed^16^. In our study population, the prevalence of DM in MSFB patients was 14.8%, which is higher than the prevalence of 8.35% (2005–2008) and 9.1% (2015–2018), respectively, in the general population in Taiwan^23^. This observation suggests that there may be a correlation between DM and the occurrence of PSFB. Hyperglycemic acidosis may impair oxidative and non-oxidative mechanisms of phagocyte fungal clearance, a major component of innate human immunity^24^. Altered microvascularization of the nasal mucosa in patients with DM also results in decreased mucociliary clearance^25^. Post-operative strict glycemic control must be instituted to prevent recurrence.

Aspergillus species are the most frequently encountered organisms in MSFB, based only on histological evidence^11^. However, only a few studies have evaluated fungal cultures of MSFB because of the low culture-positive rates resulting from poor viability of the fungal hyphae. Liu et al. collected 669 samples from PSFB for microbial cultivation, and fungi were discovered in 151 (22.6%) samples. Among them, Aspergillus spp. (72.8%) was the most prevalent fungal species^5^. In our study, fungal culture was performed in 211 MSFB cases (171 with IH and 40 without IH); it yielded a 13.74% positivity rate. *Aspergillus* spp. was the most dominant species (13 in IH group and 1 in non-IH group); however, there was no significant difference in the Aspergillus culture-positivity rate between these two groups. The correlation between the fungal species of MSFB and IH on CT scan images remains unclear. Future studies utilizing next-generation sequencing for identifying fungal species may help clarify the exact mechanism.

This study has several limitations that warrant consideration. First, we only enrolled patients who underwent ESS for PSFB; this may have introduced some degree of selection bias. Second, this study had a retrospective case–control design. We defined patients with adjacent odontogenic infection based on CT findings and related medical records; we could not determine cases with endodontic treatment and the disease course of the dental problem. Third, information regarding microbiology was not available for most patients in this study; different species of fungi may demonstrate different CT imaging features. A large-scale prospective study is thus needed for further information.

## Conclusion

In this cohort, the highest female-to-male ratio for MSFB at 51–60 years corresponded to the age of menopause. Patients with MSFB without IH were more likely to have DM, no female predominance, adjacent maxillary odontogenic pathology, and partial opacification of the sinus, compared to those with IH. These clinical features may aid earlier identification of MSFB without IH.

## Data Availability

All data described in the study has been presented in the manuscript. The datasets analyzed are available from the corresponding author on reasonable request.
